# Nurturing Grandchildren With Down Syndrome: A Qualitative Study on Grandparents’ Needs Using Digital Tools^†^

**DOI:** 10.3389/fpsyg.2021.661205

**Published:** 2021-09-13

**Authors:** María C. Sánchez Gómez, Rocío Martín-Sevillano, María V. Martín-Cilleros, J. J. Mena Marcos, Francisco J. García-Peñalvo

**Affiliations:** ^1^Department of Didactics, Organization and Research Methods, University of Salamanca, Salamanca, Spain; ^2^Department of Computer Science and Automation, University of Salamanca, Salamanca, Spain

**Keywords:** grandparents, Down syndrome, grandchildren, needs, family, qualitative investigation

## Abstract

Grandparents who have grandchildren with disabilities are an underrepresented group in existing research related to the field. This qualitative phenomenological study’s general purpose is to analyze, from a personal perspective, the situations and needs of grandparents who have grandchildren with Down syndrome. The participants’ ages range from 65 to 85, and the ages of their grandchildren with Down syndrome range from 3 to 21 years. All participants had one grandchild with a disability, except for two, who each had two. A sociodemographic questionnaire was administered, and individual interviews were conducted, using open questions, through phone and/or video calls. An analysis of the participants’ speech was carried out, which implied the development of a system of meta-categories and categories. This analysis was developed manually, given the COVID-19 environment. The results indicate a substantial change from negative feelings caused by the knowledge of the diagnosis to feelings related to positive experiences expressed currently. The participants see themselves as a fundamental source of support (informal, instrumental, practical, social, emotional, and economic) for their families and, mainly, for their grandchildren with Down syndrome. A need for information and training was observed when the grandparents talked about first being informed of the diagnosis and their concerns about the future of these grandchildren and their siblings. They made social demands, such as greater government involvement or more significant opportunities to access resources and rights for their grandchildren. The results are discussed, as are possible future research directions.

## Introduction

In recent years, social changes have transformed traditional family models (Muñoz et al., 2015). Today, grandparents play a different role than they did decades ago due to, among other reasons, difficulties reconciling work and family life, as well as an increasing tendency to have fewer grandchildren. In addition, they share more years of their lives with their grandchildren, creating a relationship that can be viewed as bidirectional (Velasco et al., 2016). Currently, older adults can be grandparents for as much as a third of their lives (Drew et al., 1998). However, despite this important reality, research on grandparents’ role is scarce. One reason is limited opportunities in the past to exercise their role in the face of shorter life expectancies and the fact that parenting was done mainly by parents. Early studies largely have not focused on grandparents’ influence on grandchildren (Velasco et al., 2016). Moreover, family members’ relationships have changed over the years. It has been observed that a social need exists to acknowledge grandparents’ role, including a desire for more closeness on the part of children and grandchildren. The relationship between grandchildren and grandparents is viewed as a unique one (Woodbridge et al., 2011).

### Grandparents and Disability

A potentially shocking moment in some grandparents’ lives is the birth of a grandchild with a disability. It is very likely that most grandparents today have limited experience with people who have disabilities, as they largely had been stigmatized, invisible, and normally excluded from society while they were growing up. Social attitudes have changed and evolved, yet many grandparents still have not considered the possibility/experience of having/caring for a grandchild with a disability (Woodbridge et al., 2011). Therefore, grandparents are experiencing new demands from the family unit (Velasco et al., 2016) and perceive a change in ways of exercising and giving meaning to their role (Sullivan et al., 2012; Muñoz et al., 2015). Some, upon the shock of learning of their grandchildren’s disability diagnosis, experience recurring feelings of guilt or a strong sense of responsibility (Lehmann et al., 2011). Because of this and other feelings, they often must reconstruct their idealized grandparental identity and, thus, readjust their expectations (Woodbridge et al., 2011). More specifically, they may give up personal life pursuits and even move to live closer to their grandchildren with a disability (Velasco et al., 2016). Such circumstances could cause grief or stress (Lee and Gardner, 2010), and possibly even double suffering created by the situation of their grandchild’s diagnosis while experiencing their children’s pain. Therefore, it is particularly important to prevent possible anxiety, anguish, and/or worries so that they do not lead to more serious consequences (Sevilla et al., 2019). Grandparents have few opportunities to learn about their grandchildren’s disabilities except through hearing secondhand information from their adult children (Lee and Gardner, 2010). However, this has been changing: Today, grandparents receive training and information through various avenues, such as disability organizations. It can be said that it is important for them to know about their grandchildren. As a study by Velasco et al. (2016) points out, grandparents’ knowledge of the type of disabilities their grandchildren have and all the information they can receive firsthand will help avoid stress produced by the absence of such knowledge. Generally, grandparents do not have direct contact with grandchildren’s doctors, educators, etc., so on certain occasions, they do not know how to react or how to help (Lee and Gardner, 2010). When grandparents are asked about their needs, they indicate that they want access to more information and assistance (p. 17). Grandparents of grandchildren with disabilities often experience increased information needs (Lee and Gardner, 2010). Some research indicates that they perceive receiving less social support than other family caregivers (Kresak et al., 2014). These situations either can be helpful or a hindrance. Similarly, the birth of a grandchild with a disability can be viewed as an element of family cohesion or stress (Lee and Gardner, 2010; Tumbarello, 2010). Some studies indicate that grandparents can be a stressor for parents facing their child’s diagnosis (Lehmann et al., 2011). Emotional support from grandparents may be significantly related to higher self-esteem among mothers, as indicated by Trute’s (2003) and Trute et al.’s (2008) research. Other studies indicate a possible weakening in the family unit if grandparents do not understand the new situation or if they cannot adapt to the new reality (Gardner et al., 2004).

Other findings indicate how internal resources, such as self-esteem or self-mastery, are associated with a lower level of stress and better mental health. The less social support grandparents have, the more stress they may experience in the early stages (Findler, 2014).

One notable aspect is how supportive grandparents are in family development with a grandchild who has a disability (Woodbridge et al., 2011). Grandparents can provide many types of support for their families, including informal, formal, economic, social, practical, and/or emotional (Poehlmann, 2003). A study by Kresak et al. (2014) demonstrated how informal support in particular can increase rates of family quality-of-life satisfaction (Cilleros and Gómez, 2016). However, Lee and Gardner (2010) found three influential variables in grandparents’ participation and support: residential proximity; parent-grandparent relations; and knowledge about their grandchildren’s disabilities.

As Muñoz et al. (2015) demonstrated, tasks related to basic activities of daily living are instrumental (e.g., rehabilitation, medical consultations, etc.), and care techniques (medication, exercise, or mobilization) are predominant with grandchildren who have a disability. According to Velasco et al. (2016), grandparents can contribute materially (shopping, errands, care, etc.) as caregivers and educators (economic support, aid with routines, etc.), providing instrumental and/or practical support. A fundamental form of support offered by grandparents is emotional (actively listening to children and grandchildren, giving advice, etc.).

### Professionals, Disability, and Family

Grandparents’ knowledge of their grandchildren’s disabilities is essential and necessary, and the literature suggests that professionals should pay attention to how families define their experience of caring for a child with a disability (Martínez, 2008; Neely-Barnes and Dia, 2008). Thus, it is necessary to consider whether planning interventions for families of children with disabilities should be the same for all family members. As Neely-Barnes and Dia (2008) noted, parents’ concerns and needs are very different from those of grandparents (Heller et al., 2000). Trute’s (2003) research suggests that it is vital that professionals in child disability services employ a family-centric model that pays attention to the importance of grandparent support. It is also essential to involve them in most of the services available.

Support groups, as Lee and Gardner (2010) stated, are viewed as a means of coping and reducing stress. Most of these groups are created in early childhood centers (Orgaz, 2009). Attendance in these groups seems to provide opportunities for grandparents to receive emotional support and guidance, thereby helping them adjust to their grandchildren’s disabilities (Ponce and Vega, 2012). They also facilitate their understanding of their grandchildren’s disabilities and how they can be supportive. In addition, they can improve grandparents’ overall well-being and provide a better parenting environment. Grandparents who have used these groups have reported that they found them useful for obtaining information about available services, providing a forum for sharing concerns, and reducing feelings of isolation (Kresak et al., 2014).

### Studies About Family and Disability

It is necessary to highlight the scarcity of research that focuses on knowing the role, situation, and needs of grandparents of grandchildren with disabilities (Miller et al., 2012). Few studies have analyzed the relationship between grandparents and grandchildren under this condition (Velasco et al., 2016), being that children’s parents generally are the protagonists in such studies (Mahoney and Perales, 2012), they provide information about grandparents’ situations to researchers; thus, the firsthand perspectives of grandparents of grandchildren with disabilities are little known or researched.

Even less research focuses specifically on Down syndrome (Hodapp, 2008; Fidler et al., 2012). In a study by Hastings et al. (2002) on grandparents’ support in families with children who have Down syndrome, the participants were 34 mothers and 27 fathers. Thus, we wish to highlight the importance and need to conduct research that focuses on grandparents’ situation, role, and needs.

Research by Fernández and Izuzquiza (2017) on parental perceptions about Down syndrome’s impact on the family exposes how greater importance is placed on the extended family, particularly grandparents, upon learning of the diagnosis. They stated the need to have better resources and support in relation to the disability, as well as to establish stable contact with other grandparents and families with similar characteristics. However, it was found that once the initial shock stage was overcome, grandparents became one of the main sources of support in the family environment, creating strong emotional bonds with their grandchildren.

### General Objective, Specific Objectives, and Research Questions

Considering the reality above, the general purpose of this research is to determine and analyze the situation and needs of grandparents of grandchildren with Down syndrome from a personal perspective. This general objective can be operationalized into the following specific objectives:

• To realize the impact that the birth of their grandchildren exerts on grandparents.
• To analyze the needs that arise after learning about the diagnosis.
• To determine whether changes occur in grandparents’ lives after the birth of their grandchildren.
• To determine whether grandparents perceive differences in existing relationships with their grandchildren with and without disabilities.
• To analyze the type of support role grandparents play within a family with a person who has a disability.
• To determine whether grandparents perceive changes in the relationships with their children since the birth of their grandchildren.
• To determine the primary source of support for grandparents of grandchildren with Down syndrome.
• To identify grandparents’ needs throughout their grandchildren’s life development.
• To discover grandparents’ current and future concerns about their grandchildren’s development.
• To analyze the influence that technology can exert on grandparents’ relationships with their grandchildren.
• To find out whether, through technology, it is possible to offer more significant resources for grandparents who have grandchildren with disabilities.

The research questions that this study aims to answer are:

– What are the needs of grandparents of grandchildren with Down syndrome?
– Do technological devices support the relationship between grandparents and their grandchildren?
– Will technology be a facilitating factor in conducting this study?

## Materials and Methods

The qualitative research methodology was used in this study. It can be characterized as inductive, understanding the context and the people under a holistic perspective, being sensitive to the effects that the researcher causes to the people under study, and understanding study participants within their frame of reference, in which all perspectives are valid. It is a humanistic method that emphasizes the validity of the research, and that all contexts and people are potential study subjects (Quecedo and Castaño, 2002). Within this qualitative research, the phenomenological method was used, specifically because the objective was to describe and interpret experiences, emotions, and/or perceptions from different participants’ unique perspectives, using their own words and/or expressions. This has implied that data collection and analysis were done simultaneously (Sampieri et al., 2014).

### Participants

Of the participants, 75% were grandmothers (women) and 25% were grandfathers (men) of grandchildren with Down syndrome. The sample comprised married couples (grandfather and grandmother), in-laws, and grandmothers. They attended a support group through contact with the Down Syndrome Foundation, thanks to Early Intervention and/or other resources that their grandchildren have used and continue to use. Purposive or convenience sampling was used with a self-selected sample. Among the participants, 66.7% are married, 25% widowed, and 8.3% separated/divorced, with all between ages 65 and 85 (*M* = 76.41). Most have higher education (58.3%), 25% have secondary education, and 8.3% have primary education and/or no education. A total of 100% of the participants are retired. A total of 50% have had contact, although not close contact, with a disability before the birth of their grandchildren; approximately 33.3% have had previous close contact; and 16.7% have had no contact at all. Most of the sample has between one and eight grandchildren (91.7%), and almost all (83.3%) have only one grandchild with Down syndrome, while 16.7% have two. More than half their grandchildren with Down syndrome are age 14 or younger (64.3%), with the rest between ages 19 and 21 (35.7%). Almost 60% of the participants live near their grandchildren. The majority (58.3%) sees their grandchildren several times a week, 33.3% daily, and 8.3% not every week. Most were informed about their grandchildren’s Down syndrome diagnosis after birth (85.7%), with only 14.3% learning about it before birth. None of the participants had another grandchild with another disability.

In the social and medical sciences, voluntary samples are standard (Sampieri et al., 2014), and this research reflects this, as it involves individuals who voluntarily agreed to participate in the study. Simultaneously, it is a self-selected sample, as the grandparents were proposed as participants and/or responded to an invitation from the Down Syndrome Foundation.

Inclusion criteria included:

• Being a grandparent
• Having one or more grandchildren with Down syndrome
• Belonging to the Down Syndrome Foundation of Madrid
• Attending or having attended a support group of the foundation
• Wishing to collaborate
• Wanting to participate
• Predisposition toward offering consent to allow the interviews to be recorded.

Finally, exclusion criteria were:

• Not being a grandparent
• Not having a grandchild with Down syndrome.

### Instruments

The instrument developed for this research comprised two parts to gather data: the first sought sociodemographic information (see Supplementary Annex I); and the second used open-ended questions/topics to gauge grandparents’ views and attitudes (see Supplementary Annex II). The second part was divided into diagnosis, family relationships, support, and needs/demands. Under *diagnosis*, feelings, communication, needs, and possible changes were discussed. Under *family relationships*, there were differences with their grandchildren with/without disability and what needs do they have. Under *support*, they were asked about the type of support role they believe they play in their families, possible changes in their relationships with their children after the diagnosis, and who or what is their most outstanding support. In the final topic, *needs/demands*, needs, changes, responses, resources, improvements, technological tools’ usability, and future demands or concerns were discussed.

### Procedure

Recruiting for the study focused on grandparents belonging to two support groups from the Down Syndrome Foundation of Madrid, one of which is Early Care (for children 0–6 years old), while the other is the School for Families (for youths ages 6–21 years). The director of the Early Attention Center, Psychologists, and Social Workers from the School for Families group was contacted and given a description of the study’s purpose and methodology. It also explained how it would follow ethical standards and require informed consent from each participant, while guaranteeing confidentiality and anonymity. Due to COVID-19 (García-Peñalvo et al., 2020, 2021), the individual interviews were conducted either through online video calls and/or by phone during March and April 2020, when all participants were confined to their homes. The importance of today’s digital technology as a means of facilitating this research during the pandemic is noteworthy. Without it, this study would not have been possible. Therefore, the technological means used by both researchers and participants was of critical importance as a helpful resource and facilitator in this research. The use of technology during the project’s first stage was essential, facilitating the study’s design, sample selection, interviews with participants, and analysis of the results.

### Data Analysis

The qualitative content analysis procedure began with an examination of the data and imposition of a structure (Sampieri et al., 2014), i.e., analyzing the interviews conducted to identify several specific themes that would later be grouped into general categories, or meta-categories. Within these categories, sub-categories were created based on existing themes. Once these were determined, a conceptual map was drawn up. An analysis was made of word frequency in each interview. Specifically, a word cloud was created with the 20 most frequent and relevant words. Concepts, themes, categories, links, and patterns present in the data were discovered, giving them meaning and allowing for interpretation to shape them according to the research objective (Sampieri et al., 2014). Coding was conducted based on the various meta-categories and categories (Sánchez-Gómez et al., 2017). To this end, each interview was analyzed in detail several times. Subsequently, participants’ experiences were described according to their perspectives, language, and expressions (Sampieri et al., 2014). Several analysis matrices were created. The first one analyzed the presence of content within each of the meta-categories. The degree of content on research, valuable resources related to that field, and personal experiences also were analyzed. The second matrix entailed creating an association of each interview’s content (textual quotations) with each meta-category. Thus, a discursive linkage and detailed analysis of each category for each interview were established (Sánchez-Gómez et al., 2017). Furthermore, the analysis required an in-depth understanding of the context surrounding the information collected to reconstruct facts and stories to link the results with available knowledge and generate a theory based on the data (Sampieri et al., 2014).

Based on a proposal by Lincoln and Guba (1985) – reflected in a scientific article by Vicario et al. (2013) – the quality assessment in qualitative research in this study comprised:

• Internal credibility validity (participant observation, data triangulation, researcher, theoretical, methodological and disciplinary, critical judgment of colleagues, collection of referential material, and participant checks)
• External transferability validity (theoretical sampling, exhaustive descriptions, and data collection)
• Dependability reliability (identification of the researcher and their role, detailed descriptions of the informants, identification and description of the analysis and data collection techniques, and delimitation of the physical, social, and interpersonal context)
• Confirmability objectivity (participant checks, mechanical data collection, and triangulation).

We used the *Qualitative Research Assessment, Elements for Critical Reading (Adapted from Critical Skills Appraisal Program (CASP) and Health Cara Libraries Unit (HCLU). Evidence-based Health Cara: An open learning resource for health care practitioners. Oxford:* CASP and HCLU, 1999) as a guide or checklist to assess the quality of our qualitative research, as it followed the order of the general research process, which includes phases such as justification, collection of information, presentation and analysis of the results, discussion, and preparation and dissemination of the final report (Vicario et al., 2013).

### Data Collection Techniques

The data collection techniques used in this study have a strong technological component. Both the first contact established with each participant and each of the audio interviews (those conducted by video call and those conducted by phone) were recorded in M4A format^1^.

To achieve the greatest completeness of the qualitative analysis (Sánchez-Gómez et al., 2017), this study used three data collection techniques: verbal (interviews); non-verbal (emotions and non-verbal communication expressed by each participant); and complementary (images, texts, books, videos, drawings, magazines, etc.).

The verbal data collection technique used different technological tools, such as computers, cell phones, tablets, recording apps, etc. The second information collection technique, non-verbal, used observation facilitated by the technological devices utilized to conduct the interviews. Finally, the complementary information collection technique entailed computerization of all the documents that various participants provided.

## Results

**FIGURE 1:**
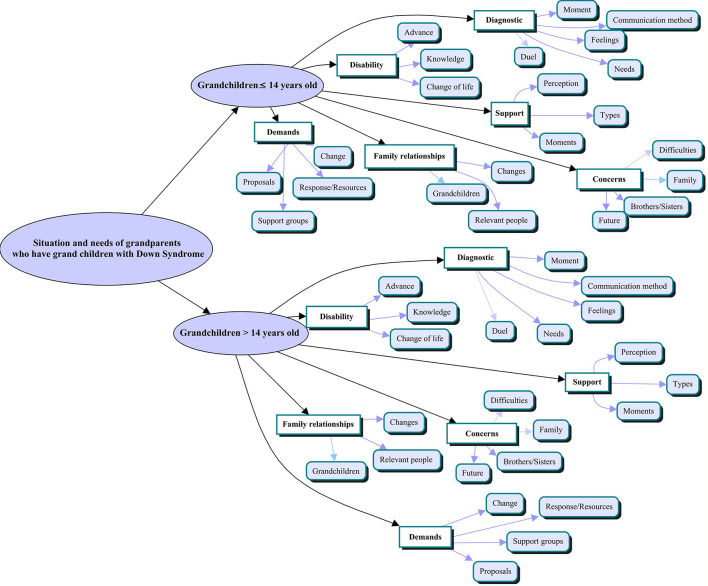
Conceptual map.

From the discourse-based categorization, the conceptual map was extracted and divided into two groups: (1) grandparents of grandchildren with Down syndrome ≤14 years old (58.33%) and (2) >14 (41.67%). Each group was organized into six meta-categories or main axes – diagnosis, disability, supports, concerns, family relationships, and demands – and 22 categories (see Figure 1).

**FIGURE 2:**
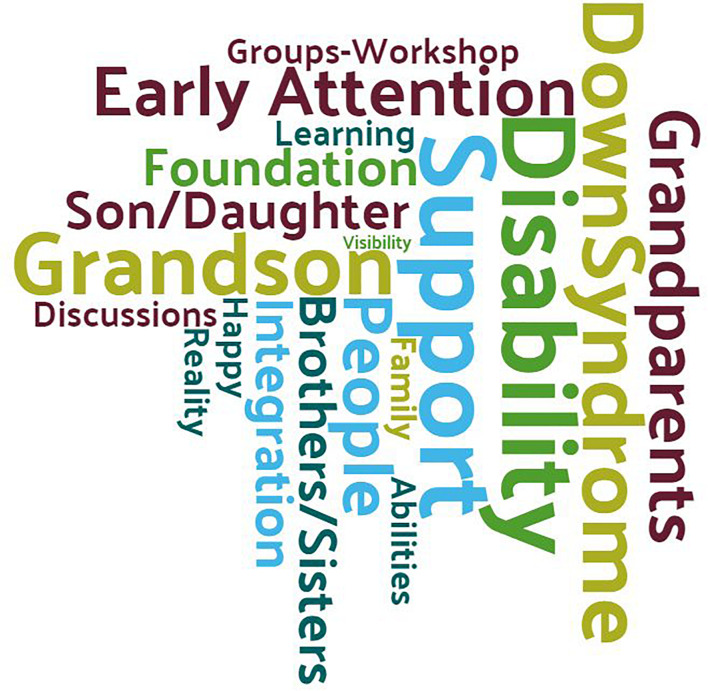
Word cloud.

**FIGURE 3:**
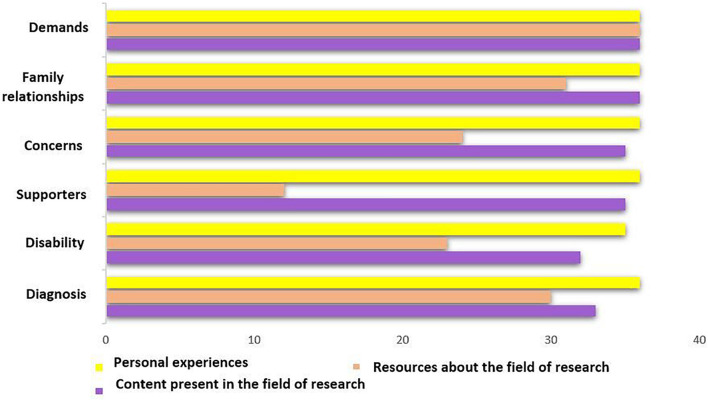
Analysis of the presence of content by meta-categories.

Down syndrome was the main theme of the interviews. Figure 2 displays the 20 most frequent words in the grandparents’ discourse. Generally, they spent a large part of the discourse talking about their grandchildren with disabilities, early intervention, disability (of their grandchildren or as a concept), the foundation, support groups, and/or the functions/support that they perform. An analysis matrix was created to analyze the presence of content from each discourse in each meta-category (see Figure 3). Three factors were analyzed in each meta-category: content present in research; resources concerning the field of research; and personal experiences. The procedure comprised analyzing each factor in each category belonging to each participant’s discourse. Each analysis factor was weighted from 1 to 3, with 1 indicating “no content of this typology is presented,” 2 indicating “content is presented, but in an ambiguous and unclear manner,” and 3 indicating “the content represented corresponds to this typology 100%.” In the six meta-categories, the score in the analysis of personal experiences (yellow) is the highest because the main objective of the research is to determine each participant’s situation and needs. In almost all the interviews, they related their experiences.

We found the most differences depending on the meta-category that we observed in the analysis of valuable resources in the field of research (pink). Under “demands,” the participants reported more useful and/or necessary resources for themselves and/or their families, including support groups and assistance from the foundation, society, and/or the government. This is reflected in participants’ statements, e.g., “That is the public environment, (where) more things and activities are offered to children with disabilities (8.33%),” “We have been shown the resources available at the foundation (8.33%),” “I signed up for all the courses (16.67%),” “Governments should do more on their part (8.33%),” “There should continue to be more resources and new resources to provide more freedom and autonomy (8.33%),” and “It would be greatly improved if it were a social duty to inform with reality (8.33%).” The categories in which the most resources are mentioned were “family relationships” and “diagnosis.” Under “family relationships,” they cited essential people who have been supportive and/or helpful in different situations as resources, e.g., “I have met many people through my grandson (8.33%),” “Now we are all united, parents, grandparents, siblings, uncles, and aunts (8.33%),” “My husband (8.33%),” “My wife (8.33%),” and “A priest friend (8.33%).” Under “diagnosis,” they presented resources related to the moment they heard about the diagnosis and, also, of human nature, e.g., “Someone who speaks to you with total sincerity and trying to help you (8.33%),” “Doctors were closer, who was not silent (8.33%),” and “That they told us differently (8.33%).” The subsequent meta-categories, in terms of resources, would be “concerns” and “disability,” e.g., “There are more and more competencies and more involvement (8.33%),” “Now they have much more freedom (8.33%),” “Now the children are very well-assisted (8.33%),” “They will look for a solution that will be the best among what is available (8.33%),” “When I am 30 (years old), there will be more resources (8.33%),” “Now they are already attended to, there are many associations that all families go to (8.33%),” and “There are many companies that provide jobs for people with disabilities (8.33%).” They cited fewer resources under “support,” as they presented and perceived themselves as a valuable and natural resource for their families, e.g., “Having the child always at home, that support (8.33%),” “I act more like a second mother than a grandmother (8.33%),” “Helping my daughter, my grandson, and being there for whatever they want (8.33%),” “We do everything for him and him (8.33%).” Referring to the content present in each meta-category of each interview, in the area of investigation (purple), very high scores near the maximums were registered, i.e., most of the analyzed content was found under “diagnosis,” “support,” “concerns,” “family relationships,” and “demands.” Most referred to substantial changes in the social and disability field from the past to the present, e.g., “Today, we can be happy that it is not like a few years ago (8.33%),” “They were as if they were isolated, mongoloid, hidden (8.33%),” “Now nothing has to do with it (8.33%),” “I felt sorry for them; now I see it differently (8.33%),” and “It has changed a lot (8.33%).” However, all meta-categories generally provided a large volume of content to analyze, as they all presented personal and particular situations and demands or needs that they perceived.

A second analysis matrix was created to establish a relationship between the different discourses with each meta-category. Two tables were created – discourses by meta-categories of grandparents of grandchildren with Down syndrome ≤14 years old and >14 – with the objective of establishing similarities and differences between the two.

### Diagnosis

Regarding the moment they were informed about their grandchildren’s disability, most stated having heard about it after birth. Only two (grandparents of two grandchildren with Down syndrome) found out during pregnancy. Most affirmed that it was an unexpected moment of significant impact. Approximately 8.33% stated that they preferred not to know beforehand to avoid making those last months of pregnancy worse. Also, 8.33% said that they would have aborted these children upon diagnosis. Communication was by phone or in person. For half the participants, it was their daughters who revealed it to them. In other cases, it was doctors and/or other health personnel (33.33%). Another family member notified another 16.67%, and another 16.67% would have preferred to be told differently. They remembered the situation as being very intense. Their first feelings upon the diagnosis were negative (anger, pain, shock, anxiety, crying, or grief). Approximately 58% reported the importance of mourning as a relevant aspect to be able to move forward, while 16.67% noted more negative feelings when the grandchild with Down syndrome was their first grandchild. Approximately 25% explained the situation generated at that moment in terms of other people close to them: They did not know whether to call or not, whether to visit them, whether to congratulate them, etc. Several participants (33.33%), when expressing their feelings, placed importance on having life experience to cope with the situation better. Half relied on faith/beliefs as a support in the face of certain events. Concerning their initial feelings after diagnosis, they all pointed out how certain feelings disappeared or changed completely over time (from negative feelings to happiness, joy, satisfaction, pride, etc.). Finally, 41.67% described having a feeling of double suffering (for the grandchild and daughter). Concerning needs expressed at the time of being informed of the diagnosis, they cited a need to be alone (8.33%), better forms of communication (P9/P10), access to medical and psychological staff (8.33%), crying and mourning (8.33%), and/or being with the family (8.33%). Several grandparents (33.33%) did not express specific needs during these first moments after hearing of the diagnosis due to the support provided by their daughter and/or their inner circle, and/or family acceptance. There was a perceived need for information about their grandchild’s disability in the early stages, but half had viewed it as unimaginable to have a grandchild with a disability in their family; thus, they were not previously informed. Referring to the diagnosis, there were no major differences between the two groups: They all reported on a moment and a situation lived and perceived years ago with similar characteristics.

### Disability

Most of the sample (75%) noted advances in society in the area of disability. They were mostly aware of changes and improvements in rights, self-determination, and freedom for people with disabilities. Others (33.33%) referred to changes in their perception of disability since the birth of their grandchild. 16.67% spoke of the importance of referring to people with disabilities as people, not merely as children with a disability. In this aspect, we found differences between the two groups—those belonging to the first group expressed and perceived changes in society. However, only 8.33% of the second group referred directly to this issue. They reported obtaining the best knowledge about their grandchildren’s disability from their daughters (41.67%), their partner (8.33%), the foundation (58.33%), and individual searches for information (33.33%). Approximately 25%, in a positive tone, indicated that they had met different people thanks to their grandchildren with Down syndrome. Some participants spoke of changes in their lives because of the birth of their grandchildren, e.g., less freedom (8.33%); skipping vacations (8.33%), and abandonment of preferences (25%). Among other aspects, 25% said they had undergone changes and have been able to integrate them into their lives. Furthermore, 16.67% perceived the changes as positive. In this aspect, both groups made similar contributions.

### Supporters

The grandparents perceived themselves as fully supportive of their family after the birth of grandchildren with a disability, assisting with their care through informal, instrumental, or practical tasks (e.g., feeding, showering, dinner, taking them to early intervention, summer, extracurricular activities, sleeping, caring, taking them to school, help with routines, etc.); social tasks (relationships with family/friends); and emotional tasks (under challenging moments in the beginning). Approximately 33.33% said they also provided economic support without providing any details beyond their willingness to provide for whatever is necessary for their grandchildren. Approximately 33.33% referred to the importance of support based on the expression “day by day.” Approximately 25% say that they temporarily have taken full care of their grandchildren to give their daughters time off. There is an individual difference in the language of the second group compared with that of the first in terms of a more supportive contribution to their grandchildren when they were younger.

### Concerns

More than half (58.33%) said that they knew they would not be alive when their grandchildren are older. There was a more significant number of direct references to their possible absence in their grandchildren’s lives in the first group than in the second. Approximately 33.33% noted that they worried about their daughters’ future, not theirs. Regarding of difficulties, 41.67% expressed concerns about problems in their grandchildren’s communication. Almost everyone in the first group pointed that their grandchildren were adolescents, which differed from the second group, whose grandchildren are no longer in that stage, so they are not worried about it. Half the participants say they are aware of their role as grandparents vs. their children’s role as parents, i.e., they realize that they are not the ones who must make crucial decisions. Approximately 25% expressed concern for the future of the siblings of their grandchildren with Down syndrome, but they all expressed concern about the future of their grandchildren with this disability. The first group expressed concerns related to adolescence, the near future, family, friendships, a future they still perceive as far away, etc. However, the second group was concerned about these grandchildren’s future after leaving school, i.e., their professional/work prospects, university education, new friendships, etc. Furthermore, 8.33% expressed concern over grandparents’ low visibility compared with parents in the realm of grandchildren with a disability. Another 8.33% expressed concern about how it is sometimes the environment that may limit opportunities for those with a disability. Some also expressed their fear of possible abuse of people with disabilities as adults. It is worth mentioning the 8.33% of the grandparents expressed a very different vision of the future when they were born compared with the one they have now. One grandparent in the second group expressed concern over how the current situation with their granddaughters with Down syndrome (21 and 20 years old) is not similar to that of other girls of the same age without disabilities. These concerns were not cited by anyone in the first group.

### Family Relationships

Around 50% of the sample noted that their Down syndrome grandchildren’s birth led to substantial changes in their lives, for the better, in terms of their relationships with their daughters. However, 16.67% said that they have not perceived any changes, and that the relationship between them and their daughters is similar to that before the birth. Regarding who they rely on the most when they need it, they cited their partner (33.33%), daughter (25%), the foundation/support group (58.33%), work colleagues (8.33%), a religious association of people with disabilities and their families (8.33%) and/or a priest (8.33%). Approximately 16.67% said they did not need any particular support. Approximately 58.33% described perceiving differences in their relationships with their grandchildren with and without disabilities vs. 41.67% who did not. Approximately 16.67% emphasized that the external environment, not the family itself, creates the most differences for their grandchildren. In this aspect, there were no notable differences between the two groups of grandparents. It is worth noting that several participants remarked on the importance of new technologies today, referring to how they create a stronger and more frequent link with their grandchildren.

### Demands

Most of the sample (75%) referred to changes over time in their information needs and how these gradually have been satisfied through their families (41.67%), the foundation (100%), or by their own means (66.66%). They all emphasized the importance of attending support meetings at the Down Syndrome Foundation of Madrid, viewing this as fundamental for their personal and social development, as well as to remain informed about Down syndrome developments and getting to know their grandchildren better (8.33%). Approximately 25% noted that the group is something unique and private – a safe place. Another 25% pointed out how important Dr. Jesús Flórez has been to them in everything related to Down syndrome. If we focus on the proposals expressed, we must highlight grandparents’ desire for greater social and governmental involvement toward people with disabilities (33.33%), more information from the beginning (16.67%), greater realization of grandparents’ role (8.33%), offering people with disabilities the same opportunities as other citizens (8.33%), treating these people with respect as the people they are (8.33%), a closer relationship with health personnel (8.33%), and an increase in available resources (8.33%). In addition, 25% referred to the importance of the coexistence of special and regular education. However, a clear difference exists between the two groups when it comes to making demands. Everyone in the first group made them, while only 19.67% in the second group made any.

## Discussion

The present study aimed to analyze the situations and needs of grandparents of grandchildren with Down syndrome. Regarding the moment they heard about the diagnosis, they reported having negative feelings, corresponding with Findler’s (2014) study. However, over time, they reported that these feelings diminished almost completely, corresponding with Velasco et al. (2016). Several described medical personnel’s role as cold/dismissive. Lehmann et al. (2011) pointed out this same aspect, referring to how these professionals may view grandparents as peripheral to the nuclear family. Some grandparents pointed to the importance of faith and belief. This corresponded with Yamashiro and Matsukura (2014) and Lehmann et al. (2011), who agreed with this idea as an important element of learning and/or a tool for coping with having a grandchild with a disability. Most reported changes in their lives since their grandchildren’s birth, and one can compare this narrative to other studies (Woodbridge et al., 2011; Miller et al., 2012; Findler, 2014; Kresak et al., 2014; Yang et al., 2018) that describe how grandparents often delayed their life goals. Some of the literature (Heller et al., 2000; Trute, 2003; Miller et al., 2012; Yang et al., 2018) corresponded with the present study’s findings in terms of types of support that grandparents provide to their families in the face of disability, i.e., informal, instrumental, practical, social, emotional, and economic. Similar to Heller et al.’s (2000) research, several grandparents noted how they provided more support to their grandchildren in the past than they do today. Similar to some participants, Miller et al. (2012) referred to how grandparents express and talk about their future death and the lack of their presence in their grandchildren’s lives as adults. Many mentioned how relationships with their daughters improved since their grandchildren’s birth, corresponding with findings by Lehmann et al. (2011), Velasco et al. (2016), and Yang et al. (2018). Results from Bruns and Foerster (2011) found that grandparents often cited their spouse as being the most supportive person in their lives. Research by Miller et al. (2012) affirmed the existence of tensions in the marriages of grandparents who have grandchildren with disabilities and possible conflicts derived from this situation. It should be noted that this research does not refer to this type of behavior, but rather the opposite. According to research by Woodbridge et al. (2011), and in line with some participants in this study, grandparents tend to use the same parenting style with grandchildren with and without disabilities, although several noted some differences. Grandparents’ concerns in this study are diverse. Among them is a clear concern for the future, thereby relating to research by Gallagher et al. (2010), Miller et al. (2012), and Yang et al. (2018). Several grandparents in the present study also pointed out their concern for the future of the siblings of their grandchildren with disabilities, corresponding with results from Lee and Gardner (2010), Miller et al. (2012), and Yang et al. (2018). Gallagher et al. (2010); Findler (2014), and Yang et al. (2018) attached importance to grandparents’ narratives that exposed their current situation as a wonderful experience, replete with happiness and a perception of personal growth since the birth of their grandchildren with this disability. These discourses are comparable to those from participants in the present study. However, some expressed their wish for more access to information related to the disability in the beginning, corresponding with Gallagher et al. (2010) and Velasco et al. (2016). However, they also emphasized the importance of having been able to educate themselves gradually. Several studies (Woodbridge et al., 2011; Velasco et al., 2016; Yang et al., 2018) highlighted grandparents’ sense of having little knowledge of and/or contact with disabilities in general, corresponding with participants in the present study. Some studies (Trute, 2003; Lee and Gardner, 2010; Kresak et al., 2014) found that grandparents place great importance on support groups with ongoing assistance, which all participants in the present study also expressed, stressing that these groups are an essential means of providing knowledge, support, and understanding.

### Methodological Limitations

Some of the limitations that this research has encountered and/or presents in relation to the participants include the following:

• Similar age range
• Similar sociodemographic characteristics
• Little cultural diversity.

Limitations related to the central theme of the research include the following:

• Insufficient and limited existing research related to the field under study.

Finally, social limitations related to this research include the following:

• COVID-19, which functioned as an impediment to recruiting participants and conducting interviews/focus groups face-to-face
• Scarce governmental participation and involvement
• Limited social consideration of the role of grandparents with grandchildren who have disabilities.

### Future Research Directions

This study comprised an important starting point for future lines of research related to grandparents, mainly considering their testimonies in the first person. Future studies could examine factors related to grandparents’ well-being, support needs, and relationships established with their children in greater depth, which would require multigenerational research. In addition, it would be interesting to conduct these studies while taking into account different cultures, races, lifestyles, and/or socio-cultural characteristics. Thus, more resources and specialized, personal, and necessary care could be offered to grandparents of grandchildren with disabilities.

It is essential to consider and keep qualitative research very much in mind to realize and develop proposed future lines of research.

## Conclusion

The results from this research are of great relevance and interest for further study of the important topic of grandparents with grandchildren who have disabilities – in this case Down syndrome. On the part of the participants, there is full recognition of the social progress of disability from the past to the present and the relevance that this change has had in their lives. Some describe the double suffering (grandchild and children) that they experienced after the birth. There is a perceived need to work more on self-determination in people with disabilities, which would prevent grandparents from worrying about the siblings of their grandchildren with Down syndrome. In examining the accounts, they also expressed a need for continuous training and information, as well as uninterrupted contact with people in a similar situation.

Thus, it can be deduced that the results presented in this research would not have been obtained without the use of modern technology. Furthermore, as some of the participants emphasized, these new technologies were important for them, as it provided a link to their grandchildren, particularly nowadays. For all these reasons, it is essential to emphasize technology’s importance in this study on several levels. Finally, it should be noted that most of the demands that grandparents made can be answered or facilitated through technological means, thereby achieving greater agility in procedures, requests, and support, as well as minimizing demands.

## Data Availability Statement

The original contributions presented in the study are included in the article/Supplementary Material, further inquiries can be directed to the corresponding author/s.

## Ethics Statement

Ethical review and approval was not required for the study on human participants in accordance with the local legislation and institutional requirements. The patients/participants provided their written informed consent to participate in this study.

## Author Contributions

MM-C and RM-S: development and theoretical approach. MS and RM-S: methodological part, sample design, elaboration of collection techniques (quality, validity, and reliability), field work with the other authors, and data analysis and results. RM-S, MS, MM-C, JM, and FG-P: assembly of the conclusion and discussion with the theoretical part, and synthesis and translation. All authors contributed to the article and approved the submitted version.

## Conflict of Interest

The authors declare that the research was conducted in the absence of any commercial or financial relationships that could be construed as a potential conflict of interest.

## Publisher’s Note

All claims expressed in this article are solely those of the authors and do not necessarily represent those of their affiliated organizations, or those of the publisher, the editors and the reviewers. Any product that may be evaluated in this article, or claim that may be made by its manufacturer, is not guaranteed or endorsed by the publisher.

## Footnotes

1 All recordings, both first contacts and entire interviews, are available to speakers in M4A format upon request.
