# Supplementary material for: Nurturing Grandchildren With Down Syndrome: A Qualitative Study on Grandparents’ Needs Using Digital Tools†
Source: Front Psychol. 2021 Sep 13;12:661205. doi: 10.3389/fpsyg.2021.661205 (PMC8473883; doi:10.3389/fpsyg.2021.661205)
Supplement: Supplementary file 1 [file Data_Sheet_1.docx]

**Nurturing grandchildren with Down Syndrome: a qualitative study on Grandparent’s needs using digital tools**

**ANNEXES**

**ANNEX I. SOCIODEMOGRAPHIC QUESTIONNAIRE**

Next, a series of sociodemographic data will be requested, solely for the purpose of determining participants’ characteristics. All data will be treated as completely confidential and anonymous.

**Please mark with an “X” the corresponding box in each case or answer manually, depending on the question.**

| **Gender** | Man |  | Woman |  |
| --- | --- | --- | --- | --- |

| **Age** |  |
| --- | --- |

|  | Single |  |
| --- | --- | --- |
|  | With partner |  |
| **Civil Status** | With domestic partner |  |
|  | Married |  |
|  | Separated/divorced |  |
|  | Widower |  |

|  | Without studies |  |
| --- | --- | --- |
| **Studies** | Primary |  |
|  | Secondary |  |
|  | Superiors |  |

| **What has been your professional performance throughout the longest period of your life?** |  |
| --- | --- |

| **Retired?** | Yes |  | No |  |
| --- | --- | --- | --- | --- |

| **Throughout your life, have you had contact with people with disabilities?** | Yes |  | No |  |
| --- | --- | --- | --- | --- |

| **If yes, what type of relationship did you have with them?** |  |
| --- | --- |

| **How many grandchildren do you have?** |  |
| --- | --- |

| **How many of your children have Down syndrome?** |  |
| --- | --- |

| **How old are they?** |  |
| --- | --- |

| **Do you live near your grandchild with Down syndrome?** | Yes |  | No |  |
| --- | --- | --- | --- | --- |

| **Approximately how many days a week do you spend with your grandchild with Down syndrome?** |  |
| --- | --- |

| **If the answer is daily, how many hours?** |  |
| --- | --- |

| **At what moment did you learn of your grandchild’s diagnosis?** | Before birth |  | After birth |  |
| --- | --- | --- | --- | --- |

| **Do you have any other grandchildren with any other type of disability?** | Yes |  | No |  |
| --- | --- | --- | --- | --- |

| **If yes, what type of disability?** |  |
| --- | --- |

**THANK YOU VERY MUCH FOR YOUR COOPERATION!**

**ANNEX II. SEMI-STRUCTURED INTERVIEW SCRIPT**

**RESEARCH ABOUT THE SITUATION AND NEEDS OF GRANDPARENTS WHO HAVE GRANDCHILDREN WITH DOWN SYNDROME, FROM A PERSONAL PERSPECTIVE**

As you know, you have been invited to participate in a research project whose objective is to analyze perceived situations and needs, at different times, among grandparents of grandchildren with Down syndrome.

The following are research questions that are viewed as most important to achieving the project’s objective.

Before I begin, I would like to thank you once again for your participation and inform you that I hope this research will help spotlight the needs of grandparents who have grandchildren with Down syndrome. **The ultimate goal is to be able to offer the best response in terms of social and human resources.**

We would like to remind you that all data and information you provide will be treated in a totally **anonymous and confidential manner.**

Having said all that, let’s start with the questions, shall we?

- I would like you to think **about when you were informed about your grandchild’s diagnosis**. I imagine that it may have been a long time ago or may have generated certain feelings that you already have forgotten. So, tell me and answer the following questions as deemed necessary: **Who told you the news, and how did you hear about it? Can you tell me how and what you felt? What did the news mean to you? What would you have needed at that time? Have you noticed any changes in your way of life since then? If so, can you tell me about them?**
- Let’s continue with the second question. To be able to answer it, you previously must have indicated in the questionnaire I provided that you have more than one grandchild. If not, you can go on to answer the next question without answering this one.

**If you have more than one grandchild**, **do you consider your relationships with your grandchildren with and without Down syndrome to be different?** If the answer is yes, **could you explain to me what needs are different or at what times/situations you noticed the differences?**

- Let’s move on to the third question. **I would like to know what kind of support you think you bring to your family**, i.e., in what areas are you most represented in your relationship with your grandchild with Down syndrome? It could be financial, social, informal, emotional, etc. In addition, I would like to know about your experience in two areas:

**Have the relationships with your children changed after having a child with Down syndrome, and if so, to what extent?**

**Whom do you rely on the most when you need help in relation to issues with your grandchild? Why?**

- Let’s proceed to the fourth question**. It is related to individually perceived needs.** I would like to know your opinion and personal experience about your needs by answering the following questions: **Have you always had the same needs? At what stage do you think you have had more needs and what kind of needs were they?**

I also would like to know **what kind of response you have received when you have presented some kind of need.** For example, it could be family, psychological, social, institutional, information, or other support. Furthermore **would you have liked or needed any other type of support? If so, what do you think you would have needed, what would you not have had, and what would have improved your situation at that time?**

- We continue with the fifth question: **How important is the use of technology for you when interacting with your grandchild? How do you think it influences your ability to seek support when needed?**

Finally, looking to the future**, can you think of any supports/resources you may need to improve your quality of life as a grandparent of a grandchild with Down syndrome?**

**THANK YOU VERY MUCH FOR YOUR COOPERATION!**
